# Attenuated Cardiac Mitochondrial-Dependent Apoptotic Effects by Li-Fu Formula in Hamsters Fed with a Hypercholesterol Diet

**DOI:** 10.1093/ecam/nep182

**Published:** 2011-02-17

**Authors:** Wei-Wen Kuo, Tsai-Ching Hsu, Mei-Haung Chain, Chao-Hung Lai, Wen-Hong Wang, Fuu-Jen Tsai, Chang-Hai Tsai, Chieh-His Wu, Chih-Yang Huang, Bor-Show Tzang

**Affiliations:** ^1^Department of Biological Science and Technology, China Medical University, Taiwan; ^2^Institute of Immunology, Chung Shan Medical University, Taiwan; ^3^Division of Cardiology, Armed Force Taichung General Hospital, Taiwan; ^4^Department of Nutrition, Taichung Veterans General Hospital, Taiwan; ^5^Graduate Institute of Chinese Medical Science, China Medical University, Taiwan; ^6^Department of Healthcare Administration, Asia University, Taiwan; ^7^Graduate Institute of Basic Medical Science, China Medical University, Taiwan; ^8^Department of Health and Nutrition Biotechnology, Asia University, Taiwan; ^9^Institute of Biochemistry and Biotechnology, Chung Shan Medical University, Taichung 40203, Taiwan; ^10^Clinical Laboratory, Chung Shan Medical University Hospital, Taiwan; ^11^Department of Biochemistry, School of Medicine, Chung Shan Medical University, Taichung, Taiwan

## Abstract

Apoptosis involves in the pathogenesis of various cardiac abnormalities. This study intends to evaluate the effects of Li-Fu formula on cardiac apoptosis induced by hyper-cholesterol diet. Twenty-four male Golden Syrian hamsters were randomly divided into Control, Cholesterol and Li-Fu formula groups. Histopathological analysis, western blotting and terminal deoxynucleotidyl transferase dUTP nick end labeling (TUNEL) assays were performed to measure the effects of Li-Fu formula on left ventricle. Significantly reduced TUNEL-positive cells and mitochondria- dependent apoptosis were observed in the left ventricle of hamsters from Li-Fu formula group compared to the Cholesterol group. Additionally, induced cardiac insulin like growth factor I receptor (IGFIR)-dependent survival pathway was detected in the Li-Fu formula group compared to the Cholesterol group. Besides, minor fibrosis, increased collagen deposition, and myofibril disarray was detected in the Cholesterol group, whereas the reductions of collagen deposition and myofibril disarray were observed in the Li-Fu formula group. This study demonstrated that Li-Fu formula not only reduced the mitochondria-dependent apoptosis and fibrosis, but also enhanced the IGF-I survival pathway in the left ventricle from high cholesterol-fed hamsters. We suggest the protective effects of Li-Fu formula on cardiac apoptosis and therapeutic potentials against cardiovascular disease.

## 1. Introduction

Hyper-cholesterol diet is known as an important risk factor that has been associated with many heart disorders including cardiac apoptosis [1, 2]. The cell apoptosis in terminally differentiated cardiomyocyte cells is a very critical pathological mechanism. Many studies have demonstrated that apoptosis may contribute to the loss of cardiomyocytes in cardiomyopathy and is regarded as a predictor of adverse outcomes in subjects with cardiac diseases or heart failure [3–5]. However, activation of IGF-I is beneficial to improve cardiac functions. Several evidences have indicated that IGF-I plays a crucial role in protection of cardiomyocytes and low IGF-I levels are associated with high risk for myocardial infarction and heart failure [6, 7]. Two major IGF-I signaling pathways, including Ras-Raf-1-Mek-ERK and 3-kinase (PI 3-kinase)-Akt pathways, have been linked to cardiac growth, proliferation, and anti-apoptotic responses [8–11].

Both Fas-dependent and mitochondrial-dependent apoptotic pathways are considered as major pathways directly to cause cardiac apoptosis [12, 13]. The recruitment of Fas-associated death domain (FADD) and pro-caspase 8 by Fas receptor oligomerization [12] initiates a death-inducing signal that results in the activation of caspase 8. The activated caspase 8 leads to cleavage of caspase 3 that executes the cell death program [14, 15]. A recent study reported the induction of Fas-dependent cardiac apoptosis in neonatal rat ventricular myocytes by predisposing hydrogen peroxide [16]. Our recent study reported the Fas-dependent cardiac apoptosis in Wistar rats that were treated with second-hand smoke [17]. Additionally, another study reported that cardiac Fas receptor-dependent apoptotic pathways were more activated in obese rats' hearts, which may provide one of the possible apoptotic mechanisms for developing cardiac abnormality in obesity [18]. The mitochondrial-dependent cell death is known as the intrinsic apoptotic pathway that is mediated by internal factors, especially in mitochondria where is the main site of action for the apoptosis-regulating proteins such as the members of B-cell CLL/lymphoma 2 (Bcl-2) family [12]. Occurrence of mitochondrial-dependent apoptosis is typically governed by contradicting the Bcl-2 family [19]. Bcl-2 is a well-known anti-apoptotic protein that can prevents cytochrome *c* release whereas Bax (Bcl-2-associated X protein) and Bad, pro-apoptotic proteins, enhance cytochrome *c* release from mitochondria into cytosol [12], which is responsible for activating caspase 9, caspase 3 and facilitates the apoptosis [20]. Numerous studies have indicated the mitochondrial-dependent cardiac apoptosis in rats that were received different treatments, including cocaine, mechanical stretch and alcohol [21–23]. Naturally, interruption of apoptosis could allow development of novel strategies to reverse or attenuate heart disorders [24].

Various western drugs such as angiotensin-converting enzyme inhibitors [25], calcium channel blockers [26], angiotensin II receptor antagonists [27] have been widely used in cardio-protective treatments. But the side effects could not be disregarded. In recent years, growing studies were performed to investigate the natural products for the cardio-protective effects that have been used as drugs or diet supplements for a long history in many medical-experiences. Various oriental herb extracts or dietary supplements have been adopted in preventing cardiac abnormality or disorders including *Fructus crataegi*, *Salvia miltiorrhiza* and *Astragali radix*. The quercetin is the main ingredient in *Fructus crataegi* that has been demonstrated as an anti-inflammatory substance by inhibiting TNF-*α* release from macrophages [28]. Recently, the *Fructus crataegi* has also been reported to have cardiac protective effect in many medical-experiences [29]. *Salvia miltiorrhiza* is known as “Danshen” and mainly composed of sodium tanshinone IIA sulfonate (STS), a derivative of tanshinone IIA. STS can reduce myocardial infarct size and prolong the survival cardiac cell in rabbit and human [30–32]. These findings support the beneficial effect of STS in protecting the heart. *A. radix* contains many isoflavones and isoflavonoids, such as formononetin, calycosin and ononin, and many saponins, such as astragaloside IV, astragaloside II, astragaloside I, and acetylastragaloside I [33]. *A. radix* has demonstrated the effects against inflammation and cardiac ischemia reperfusion injury and has been suggested the protective effect on heart [34–36].

In the current study, to understand the effects of Li-Fu formula on cardiac protection, we examined not only the Fas-dependent and mitochondrial-dependent apoptosis but also the IGF-I survival pathway in the cardiac tissues from hamsters that were fed with a hypercholesterol diet. We suggested the cardiac protective effect of Li-Fu formula by activating the IGF-I survival pathway and inhibiting the cardiac mitochondria-dependent but not Fas-dependent apoptosis.

## 2. Methods

### 2.1. Animals and Diet

A total of 24 male Golden Syrian hamsters weighting 145–170 g at the age of 8 weeks were purchased from National Laboratory Animal Center, Taipei, Taiwan, and used in this experiment. Hamsters were acclimatized for 2 weeks while receiving free access to water and were fed chow diet (Lab Diet 5001; PMI Nutrition International Inc., Brentwood, MO, USA) ad libitum. The hamsters were then randomized into three groups as control, cholesterol and Li-Fu formula groups and switched to experimental diets. The control, cholesterol and Li-Fu formula groups received chow diet, chow diet with 0.2% cholesterol (Sigma, St Louis, MO, USA), and chow diet with 0.2% cholesterol and 2% Li-Fu formula for 8 weeks, respectively. The Li-Fu formula was firstly created and provided by Dr. Li-Fu Chen, China Medical University, Taichung, Taiwan. To make Li-Fu formula, every component of desired weight was crushed and mixed with a blender, then placed in distilled water (1000 ml) and boiled for 1 h under reflux. The resultant solution was divided into several parts and stored in a –80°C freezer for further use. Formulation and calculated composition of experimental diets were shown in Table 1. The ambient temperature was maintained at 25°C. Diets were prepared weekly and stored at –80°C. All experimental procedures were performed according to the NIH Guide for the Care and Use of Laboratory Animals. All protocols were approved by the Institutional Animal Care and Use Committee of China Medical University, Taichung, Taiwan. Food intake and food spillage were measured daily, and body weight was recorded every 3 days. 

### 2.2. Transferase-Mediated dUTP Nick End Labeling (TUNEL)

After the hearts were excised, the hearts were soaked in formalin and covered with wax. In heart tissues, the 3-*μ*m thick paraffin sections were cut from formalin-fixed, paraffin-embedded tissue blocks. The sections were deparaffinized by immersing in xylene, re-hydrated, and incubated in phosphate-buffered saline with 2% H_2_O_2_ to inactivate endogenous peroxidases. The sections were then incubated with proteinase K (20 *μ*g/ml), washed in phosphate-buffered saline, and incubated with terminal deoxynucleotidyl transferase for 90 min and fluorescein isothiocyanate-dUTP for 30 min at 37°C using an apoptosis detection kit (Roche Applied Science, Indianapolis, IN, USA). Samples were analyzed in a drop of PBS under a fluorescence and UV light microscope at this state by an excitation wavelength in the range of 450–500 nm and detection in the range of 515–565 nm. The number of TUNEL-positive cardiac myocytes was determined by counting 3 × 10^5^ cardiac myocytes. All morphometric measurements were performed by at least two independent individuals in a blinded manner.

### 2.3. Tissue Extraction

Cardiac tissue extracts were obtained by homogenizing the left ventricle samples in a PBS buffer (0.14 M NaCl, 3 mM KCl, 1.4 mM KH_2_PO_4_, 14 mM K_2_HPO_4_) at a ratio of 100 mg tissue/0.5 ml PBS for 5 min. The homogenates were placed on ice for 10 min and then centrifuged at 12 000 g for 30 min. The supernatant was collected and stored at –70°C for further experiments.

### 2.4. Electrophoresis and Western Blot

The tissue extract samples were prepared as described in Methods section. Sodiumdodecyl sulfate-polyacrylamide gel electrophoresis was performed with 10% polyacrylamide gels. Protein concentration was determined using a BioRad Protein Assay (BioRad Laboratories, Hercules, CA, USA) and were quantified by absorbance at 595 nm using a spectrophotometer (Beckman Coulter, Palo Alto, CA, USA). The samples were electrophoresed at 140 V for 3.5 h and equilibrated for 15 min in 25 mM Tris–HCl, pH 8.3, containing 192 mM glycine and 20% (V/V) methanol. Electrophoresed proteins were transferred to nitrocellulose membranes (Amersham, Hybond-C Extra Supported, 0.45 *μ*m pore size) with a Bio-Rad Scientific Instruments Transphor Unit at 100 mA for 14 h. Nitrocellulose membranes were incubated at room temperature for 2 h in blocking buffer containing 100 mM Tris–HCl, pH 7.5, 0.9% (w/v) NaCl, 0.1% (v/v) fetal bovine serum. Antibodies including Fas, Bad, Bcl-2, cytochrome *c*, caspase 8, IGFI receptor, PI3K, p-AKT, AKT, *α*-tubulin (Santa Cruz Biotechnology, Santa Cruz, CA, USA), cleaved caspase 3 (Asp175) and cleaved caspase 9 (Asp315) (Cell Signaling, MA, USA) were diluted to 1 : 200 in antibody binding buffer containing 100 mM Tris–HCL, pH 7.5, 0.9% (w/v) NaCl, 0.1% (v/v) Tween-20 and 1% (v/v) fetal bovine serum. Incubations were performed at room temperature for 3.5 h. The immunoblots were washed three times in 50 ml blotting buffer for 10 min and then immersed in the second antibody solution containing horseradish peroxidase (HRP) conjugated goat anti-hamster IgG (Promega Corp., Madison, WI, USA) for 1 h that was diluted 1000-fold in binding buffer. The immunoblots were then washed in blotting buffer for 10 min three times. Pierce's; Supersignal West Dura HRP Detection Kit (Pierce Biotechnology Inc., Rockford, IL) was used to detect antigen–antibody complexes. The blots were scanned and quantified by densitometry (Appraise, Beckman–Coulter, Brea, California, USA).

### 2.5. Masson Trichrome Staining

The hearts of animals were excised, and were soaked in formalin and covered with wax. Slides were prepared by deparaffinization and dehydration. They were passed through a series of graded alcohols (100, 95 and 75%), 15 min each. The slides were then dyed with Masson trichrome. After gently rinsing with water, each slide was then soaked with 85% alcohol, 100% alcohol I and II for 15 min each. At the end, they were soaked with Xylene I and Xylene II. Photomicrographs were obtained using Zeiss Axiophot microscopes.

### 2.6. Statistical Analysis

All the statistical analyses were performed using SPSS 10.0 software (SPSS Inc., Chicago, IL). Three independent experiments were repeated. Statistical analyses were performed using the analysis of variance plus posterior multiple comparison test to test the difference. The data between two experimental animal groups was compared by Student's *t*-test for two independent samples. In all cases, a difference at *P* < .05 was considered statistically significant.

## 3. Results

### 3.1. TUNEL-Positive Apoptotic Cells of Cardiac Tissues

**Figure 1:**
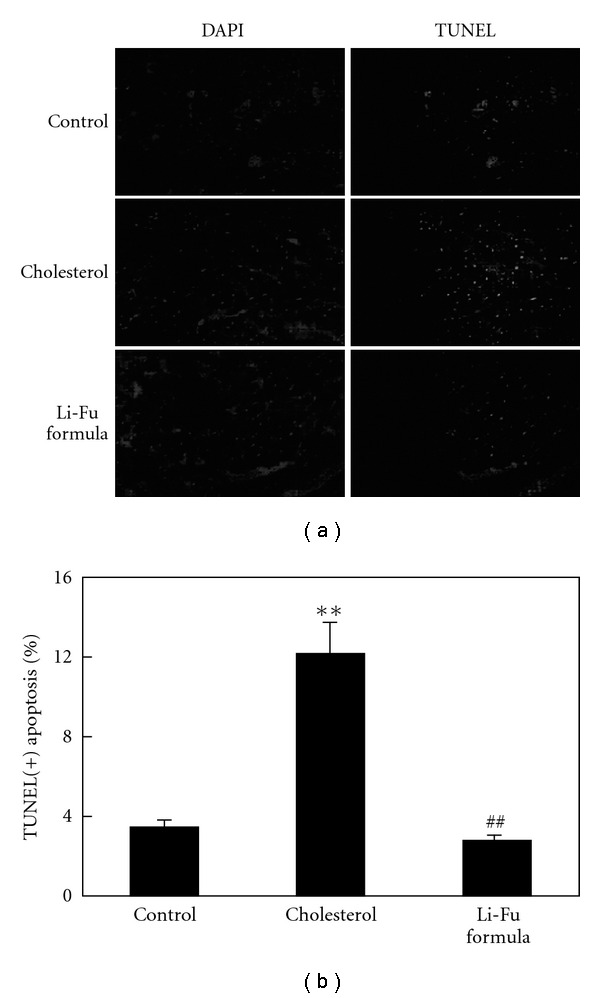
(a) Representative stained apoptotic cells of cardiac sections from left ventricles in hamsters of Control, Cholesterol and Li-Fu formula groups were measured by staining with 4′,6-diamidino-2-phenylindole (DAPI) (left panels) and TUNEL assay with dark background (right panels). The images were magnified by 400×. (b) Bars present the percentage of TUNEL positive cells relative to total cells (6 rats × 30 scope field count in each group). **P < .01, significant differences between Control and Cholesterol group. ^##^ *P* < .01, significant differences between Cholesterol and Li-Fu formula groups.

To investigate the effect of Li-Fu formula on hyper-cholesterol diet induced apoptosis in cardiac cells, we examined the apoptosis-positive cardiac cells in the excised hearts of hamsters from Control, Cholesterol and Li-Fu formula groups by TUNEL assay. We found that left ventricle stained with TUNEL assay showed increased TUNEL-positive cardiac cells in the Cholesterol group compared to the Control group (Figure 1(a)). Notably, significantly reduced TUNEL-positive cardiac cells were found in the left ventricle of hamsters from Li-Fu formula group compared to the Cholesterol group (Figure 1(a)). The percentage of TUNEL-positive cardiac cells was calculated and the quantified results were shown in Figures 1(b). 

### 3.2. Effect of Li-Fu Formula on Cardiac Fas and Mitochondrial-Dependent Apoptotic Pathway

**Figure 2:**
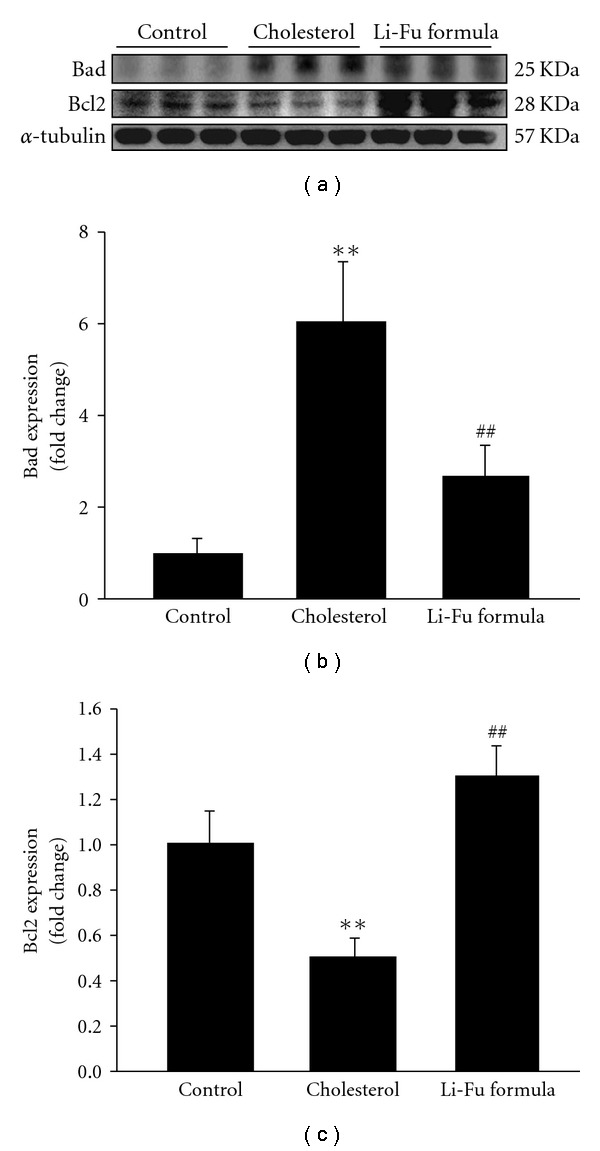
(a) The representative protein products of Bad and Bcl-2 extracted from the left ventricles of excised hearts in hamsters of Control, Cholesterol and Li-Fu formula groups were measured by western blotting analysis. ((b) and (c)) Bars represent the relative protein quantification of Bad and Bcl-2 on the basis of *α*-tubulin. All bars indicate mean values ± SD (*n* = 6 in each group). **P < .01, significant differences between Control and Cholesterol group. ^##^ *P* < .01, significant differences between Cholesterol and Li-Fu formula groups.

**Figure 3:**
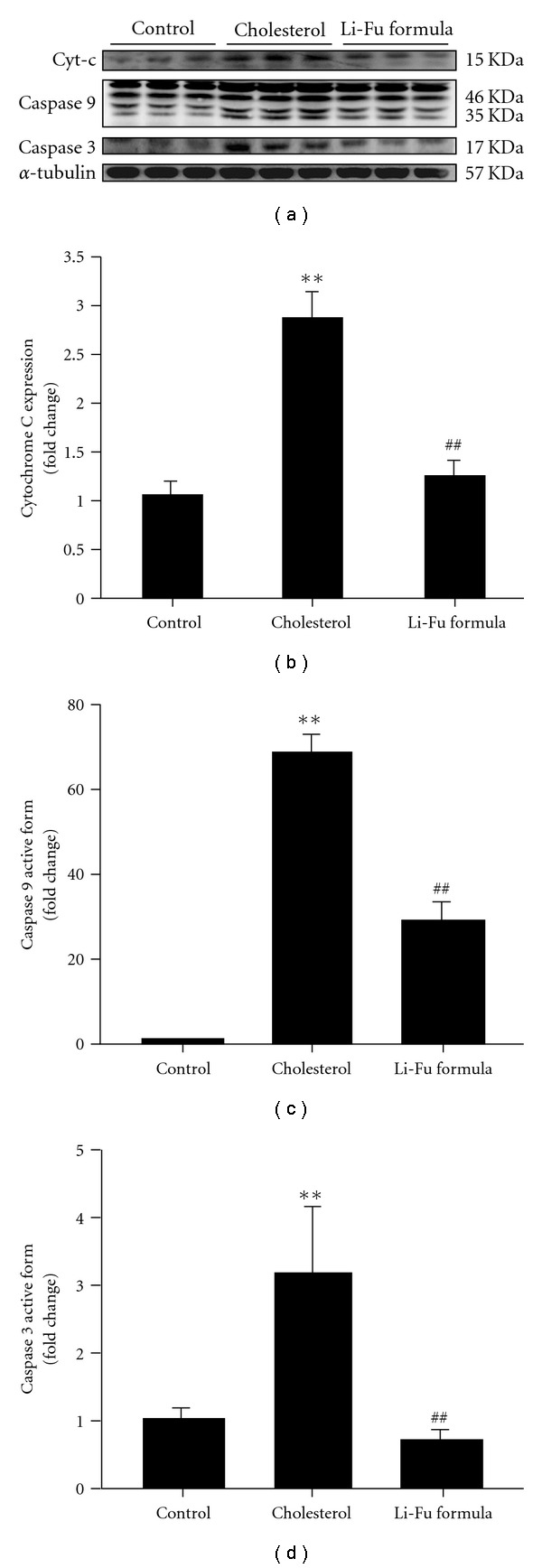
(a) The representative protein products of cytosolic cytochrome *c,* caspase 9 and caspase 3 extracted from the left ventricles of excised hearts in hamsters of Control, Cholesterol and Li-Fu formula groups were measured by western blotting analysis. ((b), (c), and (d)) Bars represent the relative protein quantification of cytosolic cytochrome *c,* caspase 9 and caspase 3 on the basis of *α*-tubulin and indicate mean values ± SD (*n* = 6 in each group). **P < .01, significant differences between Control and Cholesterol group. ^##^ *P* < .01, significant differences between Cholesterol and Li-Fu formula groups.

**Figure 4:**
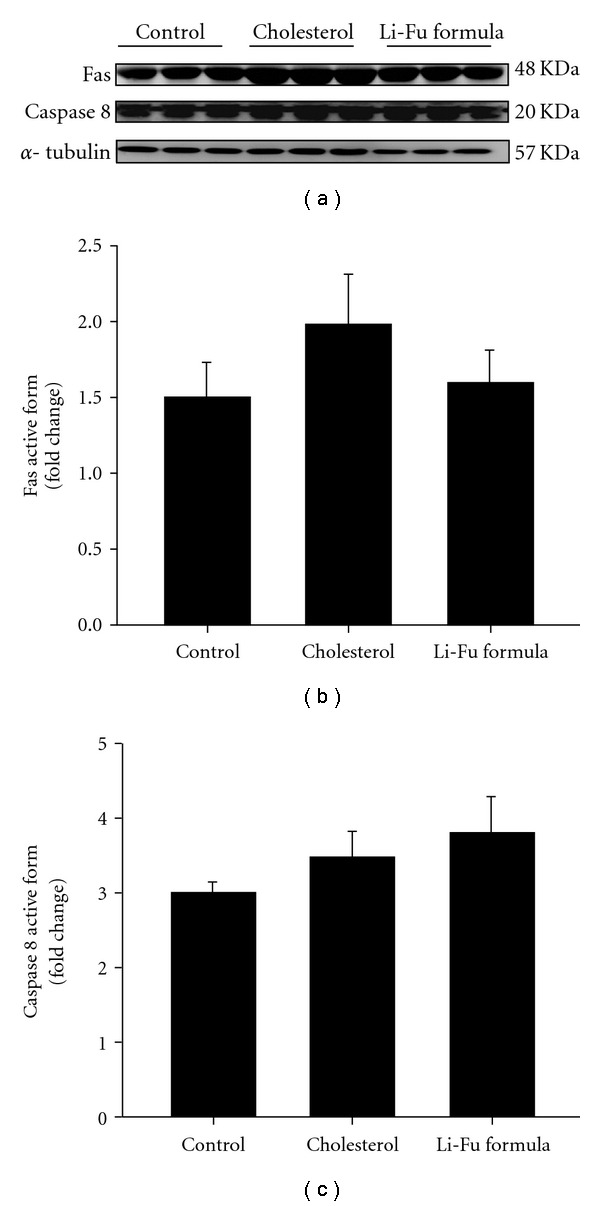
(a) The representative protein products of Fas receptor and caspase 8 extracted from the left ventricles of excised hearts in hamsters of Control, Cholesterol and Li-Fu formula groups were measured by western blotting analysis. ((b) and (c)) Bars represent the relative protein quantification of Fas receptor and caspase 8 on the basis of *α*-tubulin, and indicate mean values ± SD (*n* = 6 in each group).

To further understand the cardiac Bcl-2 family in mitochondrial-dependent apoptosis, we examined the protein levels of the Bcl-2 family (Bad, Bcl-2) in the excised hearts of hamsters from Control, Cholesterol and Li-Fu formula by western blotting. The expression of Bad protein was significantly increased in the Cholesterol group whereas anti-apoptotic proteins Bcl-2 were significantly reduced in the Cholesterol group (Figure 2). Notably, we found that significantly reduced Bad and increased Bcl2 protein expression in the excised hearts of hamsters from Li-Fu formula group compared to the Cholesterol group (Figure 2). Quantified protein level and the fold changes were shown in Figures 2(b) and 2(c). Moreover, the presence of cytosolic cytochrome *c* (cytochrome *c* release from mitochondria), caspase 9 and caspase 3 in the excised hearts of hamsters from Control, Cholesterol and Li-Fu formula were analyzed by western blotting. The protein levels of cytochrome *c*, caspase 9 and caspase 3 were significantly increased in the Cholesterol group compared to the control group. In contrast, significantly reduced cytochrome *c*, caspase 9 and caspase 3 were detected in excised left ventricle of hamsters from Li-Fu formula group compared to the Cholesterol group (Figure 3). Quantified protein levels and the fold changes of cytochrome *c*, activated caspase 9 and activated caspase 3 were shown in Figures 3(b), 3(c), and 3(d), respectively. To identify the downstream components of cardiac Fas-dependent apoptotic pathway, we examined the protein levels of Fas and caspase 8 in the excised hearts of hamsters from Control, Cholesterol and Li-Fu formula by western blotting. However, no significant variation was observed among the three groups of hamsters (Figure 4). Quantified protein levels of Fas and caspase 8 were revealed in Figures 4(b) and 4(c), respectively. 

### 3.3. Effect of Li-Fu Formula on Cardiac IGFIR-Dependent Survival Signaling Pathway

**Figure 5:**
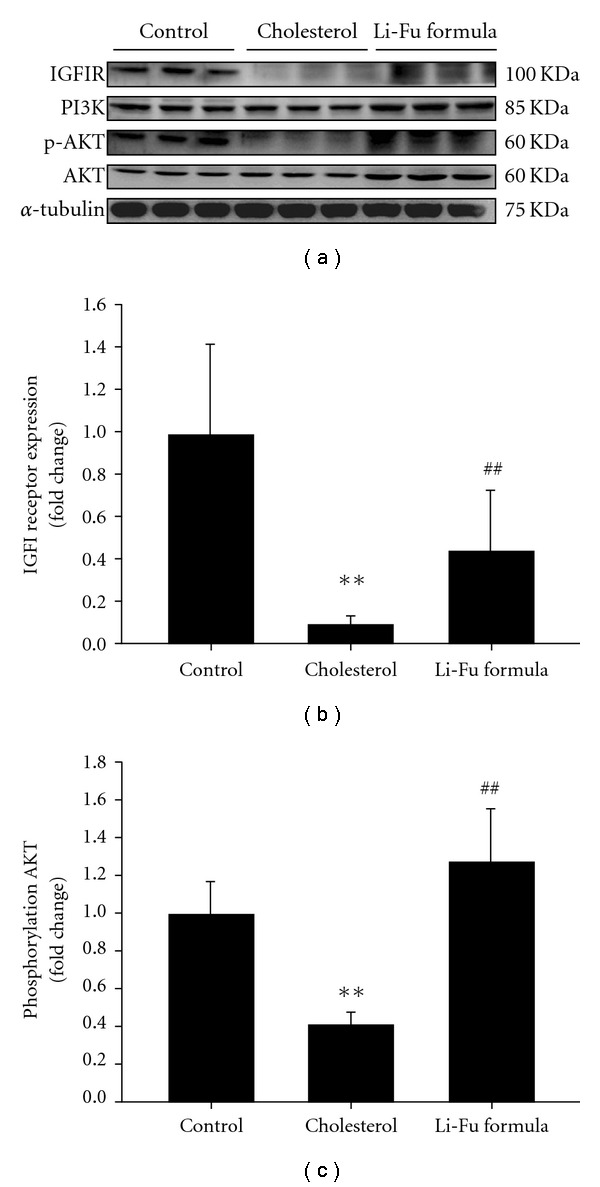
(a) The representative protein products of IGFIR, PI3K, p-AKT and AKT extracted from the left ventricles of excised hearts in hamsters of Control, Cholesterol and Li-Fu formula groups were measured by western blotting analysis. ((b) and (c)) Bars represent the relative protein quantification of IGFI receptor and phosphorylated AKT on the basis of *α*-tubulin, and indicate mean values ± SD (*n* = 6 in each group). **P < .01, significant differences between Control and Cholesterol group. ^##^ *P* < .01, significant differences between Cholesterol and Li-Fu formula groups.

To identify the effect of Li-Fu formula on cardiac IGFIR dependent survival pathway, we examined the protein levels of IGFI receptor, PI3K, AKT and p-AKT in the excised hearts of hamsters from Control, Cholesterol and Li-Fu formula by western blotting. The protein levels of IGFI receptor, PI3K, AKT and phosphorylated AKT were significantly reduced in the left ventricle of hamsters from Cholesterol group compared to Control group (Figure 5(a)). Notably, we found significantly increased IGFI receptor, PI3K, AKT and phosphorylated AKT in the excised left ventricles of hamsters from Li-Fu formula group compared to the Cholesterol group (Figure 5(a)). Quantified protein levels and the fold changes of IGFI receptor and phosphorylated AKT were shown in Figures 5(b) and 5(c), respectively. 

### 3.4. Cardiac Fibrosis Changes

**Figure 6:**
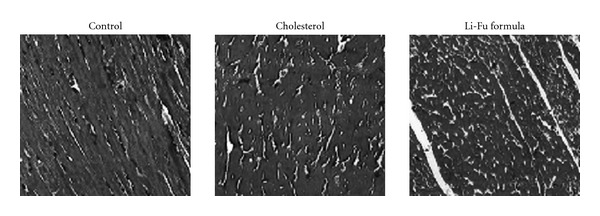
Representative histopathological analysis of cardiac tissue sections with Masson-trichrome staining (fibrosis) in hamsters of Control, Cholesterol and Li-Fu formula groups. The images of myocardial architecture were magnified 100×.

To examine the effect of Li-Fu formula on cardiac fibrosis in hamsters, we performed a histopathological analysis of ventricular tissue with Masson-trichrome staining. Hearts stained with Masson-trichrome showed minor fibrosis, increased collagen deposition, and myofibril disarray in Cholesterol group compared to the Control group (Figure 6). Notably, significantly reduced collagen deposition, and myofibril disarray was observed in the Li-Fu formula group compared to the Cholesterol group (Figure 6). 

## 4. Discussion

Apoptosis is known to play crucial roles and regarded as a predictor in various cardiac diseases or heart failure [3, 5, 24]. Since the side effects of western drugs in treatment of cardiac diseases cannot be avoided, the investigation of natural products or dietary supplements to protect cardiac abnormality and injury is essential. This study firstly demonstrated the protective effects of Li-Fu formula on cardiac apoptosis in hamsters that were fed with a high-cholesterol diet. Our results revealed that Li-Fu formula not only reduced the mitochondria-dependent apoptosis and fibrosis, but also enhanced the IGF-I survival pathway in the left ventricle from high cholesterol-fed hamsters.

**Figure 7:**
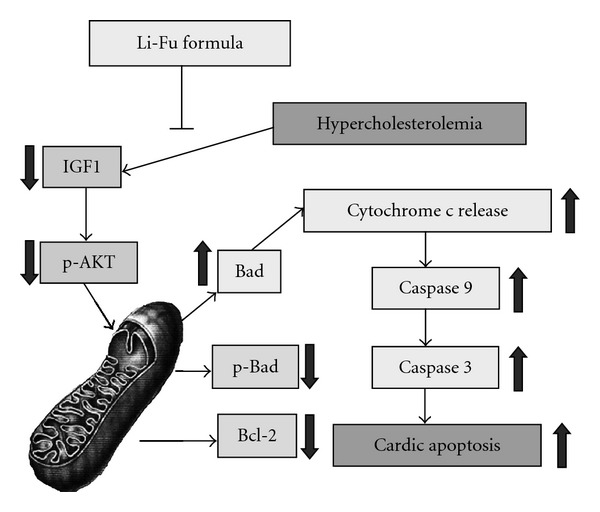
The effects of Li-Fu formula on hypercholesterolemia-induced mitochondrial-dependent apoptotic pathway in hamsters. Our proposed integrative hypothesis indicates that cardiac mitochondrial-dependent apoptotic pathway is more active under hypercholesterolemia, whereas IGF-PI3K-Akt is down regulated. The decreased IGF-1, phosphorylated Akt, decreasaed Bcl2, phosphorylated Bad, increased pro-apoptotic Bad, increased cytochrome *c* release, increased activated-caspase 9 and increased caspase 3 were detected in the heart of hypercholesterolemia hamaster. The arrow represents its increasing or decreasing under the condition of hypercholesterolemia. All these changes can be suppressed by Li-Fu formula treatment.

Excess dietary cholesterol is responsible for the hypercholesterolemia that has been recognized as the significant risk factor to cause cardiac injury or diseases [37]. A recent study reported that hypercholesterolemia reduced endomyocardial coronary flow reserve, capillary density and induced capillary endothelial cell apoptosis in minipigs [38]. Another study indicated the induction of coronary atherosclerosis and myocardial fibrosis in rabbits fed with hypercholesterol diet [39]. Since the increased TUNEL-positive cardiac cells were detected in hypercholesterol rabbits [40], the cardiac apoptotic pathway induced by hypercholesterol diet is still unclear. In our experimental findings, mitochondrial-dependent cardiac apoptosis was significantly increased in excised left ventricle from hamsters fed with a high-cholesterol diet (Figure 7). However, no variation of Fas-dependent apoptotic components was detected in all hamsters. Therefore, we suggested the Fas-independent but mitochondrial associated cardiac apoptosis in hamsters that were fed with a high-cholesterol diet. 

IGF-I is a survival factor in cardiomyocytes that activates the PI3K-Akt/PKB pathway [9]. The IGF-I can bind to cell surface IGF-IR and cause a conformational change resulting in activation of its tyrosine kinase domain and autophosphorylation of tyrosine and serine residues [41, 42]. Once activated, the IGF-IR causes phosphorylation of various proteins including insulin receptor substrate (IRS)-1 and IRS-2 [43]. Recent reports have indicated that increased Bcl-xL level in mitochondrial was observed in IGF-I pretreated rats and cardiac-specific IGF-I overexpression is anti-apoptotic [44] whereas increased apoptosis after myocardial infarction was observed in IGF-I deficient mice [45]. As consistent with our experimental results, the significant reduction of IGF-IR pathway associated components was detected in the excised ventricle from hamster of Cholesterol group. In contrast, significantly increased IGFIR signaling components and reduced cardiac apoptosis were observed in the Li-Fu formula group. These findings suggested that Li-Fu formula attenuates the cardiac apoptosis and facilitates the IGF-IR cardiac survival pathway (Figure 7).

The use of dietary supplements or herbal medicine for the treatment of various disorders including heart diseases has a long and extensive history. In the world, more than half of the population relies on traditional medicine for therapeutic needs either by stewing or solution extracting [46, 47]. Although the precise mechanism of most herbal medicine or dietary supplement has not been fully understood, the experience of the traditional use over the years cannot be neglected. The Li-Fu formula, initially created by Dr Li-Fu Chen, China Medical University, Taichung, Taiwan, is composed of Celery, Black fungus, Mushroom, *Saliva miltiorrhiza*, *Crataegi cuneata* and *A. radix* as shown in Table 1. Li-Fu formula is not only a formula of traditional herbal medicines but also routinely used as dietary supplements. Additionally, the ingredient of Li-Fu formula from different batches has the very similar compositions that are in acceptable inaccuracy. Moreover, Li-Fu formula from different batches has the same effects on cardiac protection. Since the major components of Li-Fu formula have been suggested to have various cardiac protective effects [26, 27, 30–32, 35, 36], the precise actions on prevention of hypercholesterolemia-induced cardiac apoptosis are still unclear. In the current study, we have demonstrated the beneficial effects of Li-Fu formula by significantly reducing cardiac fibrosis, mitochondrial-dependent apoptosis and activating of IGF-IR survival pathway and suggest the potential of Li-Fu formula on cardiac protection.

Taken together, we demonstrated the mitochondria-dependent but not Fas-dependent cardiac apoptosis in hamsters fed with hypercholesterol diet, and suggested the cardio-protective effect on activating the IGF-IR survival pathway by treatment of Li-Fu formula. Although the mechanism of Li-Fu formula on cardio-protective effects is not completely understood because of the limitation of research methodology and complexity of Chinese traditional medicine, further works are merited to be performed with single compound such as STS, quercetin, formononetin, calycosin and ononin to re-examine the effects of these compounds.

## Funding

China Medical University, Taichung, Taiwan (CMU96-100).

## Figures and Tables

**Table 1:** Formulation and calculated composition of experimental diets.

Ingredients (% wt/wt)	Control	Cholesterol	Li-Fu formula
Chow diet, (Rodent 5001)	99.5	99.3	97.3
Soybean oil	0.5	0.5	0.5
Cholesterol	0	0.2	0.2
Li-Fu formula			
Celery^(a)^	0	0	0.05
Black fungus^(b)^	0	0	0.646
Mushroom^(c)^	0	0	0.848
*Saliva miltiorrhiza*	0	0	0.152
*Crataegi cuneata*	0	0	0.152
*A. stragali radix*	0	0	0.152

^(a)^Celery is also known as *Apium graveolens*; ^(b)^Black fungus indicates Wood ear, or pinyin: mù ěr, lit. “wood ear" or “tree ear" are commonly sold in Asian markets as dietary supplement; ^(c)^The standard for the name “mushroom" is the cultivated white button mushroom, *Agaricus bisporus*.
